# Evaluation and Interventional Management of Pain After Vertebral Augmentation Procedures

**DOI:** 10.7759/cureus.1061

**Published:** 2017-02-28

**Authors:** Jesse Hatgis, Michelle Granville, Robert E Jacobson

**Affiliations:** 1 Larkin Hospital, Nova Southeastern University School of Osteopathic Medicine; 2 Miami Neurosurgical Center, University of Miami Hospital

**Keywords:** vertebral compression fracture, vertebroplasty, kyphoplasty, compression fracture, vertebral augmentation

## Abstract

**Introduction:**

A small subset of patients who underwent successful vertebral compression fracture (VCF) augmentation procedures may develop subsequent pain requiring spinal injections. In a retrospective analysis, we determined whether the pain was related to the original fracture site or to another area within the lumbar or thoracic spine. The pain occurred either at the same/adjacent level and/or non-adjacent level as the VCF. Interventional treatments primarily targeted the facet joints, specifically in the form of facet joint blocks and/or radiofrequency ablation to the medial branches. The pattern of facet injections relative to the original fracture level was studied. Additionally, the elapsed time between the vertebral augmentation and the subsequent interventional blocks was also evaluated.

**Methods:**

A total of 56 patients sustained VCFs. 12 of these patients underwent interventional procedures after vertebral augmentation procedures. The level(s) of same/adjacent level and non-adjacent level pain were determined via physical examination and/or imaging studies. These levels were subsequently treated with interventional procedures primarily focused on the facet joints. The time period of the injections varied from two weeks status post-vertebral augmentation to as late as 304 weeks (5.8 years) status post-vertebral augmentation.

**Results:**

We performed 25 vertebral augmentation procedures on these 12 patients. 15 lumbar, eight lower thoracic, and two mid-thoracic VCFs were augmented. 9/14 cases of blocks included those performed at non-adjacent levels, whereas 5/14 cases of blocks were performed only at the same and/or adjacent levels as the VCF. For the events in which thoracic VCFs were augmented, 6/7 (or 86%) had developed non-adjacent level pain in areas of the lumbar spine.

The time from vertebral augmentation procedure to subsequent pain procedure ranged from two weeks to five plus years. The average time elapsed was 83 weeks. Only one case required blocks performed within the first six weeks after vertebral augmentation. In this case, the blocks included those at non-adjacent levels. A total of 4/12 cases (33%) had a block within 12 weeks of the original vertebral augmentation procedure.

Lumbar spine imaging showed that at least 9/12 patients had pre-existing significant lumbar pathology at the time of fracture treatment. This may have contributed to the later development of pain.

**Conclusion:**

Pain after a successful vertebral augmentation is typically non-acute (i.e., beyond six weeks). Mechanisms other than the primary VCF are usually responsible for non-adjacent level pain, which are present a majority of the time on reviewing the patients' diagnostic studies. These mechanisms usually take many weeks to develop and subsequently elicit pain that requires additional interventional pain procedures. In our study, the pain is usually related to the pre-existing degenerative spondylosis and stenosis rather than the fracture site. This study shows that the facet joints in closely related lumbar degenerative changes are the cause of pain in this patient group. These procedures should be explored with pain after vertebral augmentation, especially in those patients with known or suspected spinal degeneration and/or poor biomechanics.

## Introduction

In 2007, Georgy reviewed a subset of patients from a larger group who underwent vertebral compression fracture (VCF) augmentation procedures and subsequently required spinal injections to reduce pain [1]. He stated that both the original compression fracture, as well as the subsequent vertebral augmentation, may result in pain either at the same level as the fracture or at an adjacent level. He chose to address this issue via mainly performing epidural steroid injections (ESIs) at unspecified levels. Alternatively, Kim, et al. (2005) primarily used facet joint blocks at the same and adjacent levels as augmented VCFs to determine the most painful spinal level of patients with multiple VCFs [2].

We performed a retrospective analysis of our patient database, reviewing all patients who underwent vertebral augmentation and later had some post-procedure pain, requiring another interventional pain procedure. We documented whether the pain was related to the original fracture site or related to another area within the lumbar or thoracic spine either at the same/adjacent level and/or non-adjacent level. The elapsed time of the pain in relation to when the fracture was treated was also evaluated. The normal post-operative period was considered six weeks. Interventional pain management targeted the facet joint rather than the epidural space as the major source of pain. Interventional treatments consisted of primarily facet joint blocks and/or radiofrequency ablation to the medial branches in the majority with several patients also having epidural or sacroiliac joint blocks. The pattern of facet injections relative to the original fracture level was then analyzed showing that pain procedures in the first six weeks post-operatively were actually the exception. The later pain was related to chronic spinal pain often delayed by many months or years due to associated general spinal degenerative changes, poor body mechanics, and lack of exercise in this age group rather than specifically as a result of the treated fracture.

Informed consent was obtained from the patients for this study. 

## Materials and methods

We analyzed the data of 56 patients who sustained VCFs. We included only those patients who underwent interventional blocks after vertebral augmentation procedures. 12 patients fulfilled this criterion. 10 of the 12 only had one interventional procedure while 2/12 had a second procedure, thus there were 14 different events. For those patients with persistent pain, we determined the level(s) of same/adjacent level and non-adjacent level pain via physical examination and/or imaging studies which included plain radiographs, computed tomography (CT), magnetic resonance imaging (MRI), and/or bone scan. We subsequently performed interventional procedures primarily focused on the facet joints. The time period of the injections varied from two weeks status post-vertebral augmentation to as late as 304 weeks (5.8 years) status post-vertebral augmentation. If the pain location after vertebral augmentation was at the same and/or adjacent levels as the previously augmented vertebra, we then treated the same and/or adjacent levels with interventional blocks, primarily at the facet joints above and below the level fractured, as well as at the level of the fracture. If patients had non-adjacent level pain, then the areas of subsequent pain generation were effectively treated with interventional blocks based on both clinical examination and radiologic studies. Office follow-ups were scheduled two and six weeks post-procedure.

## Results

Please refer to Table 1. 

**Table 1:** Characteristics of Events * One of two events for a patient; ** One of two events for another patient B/L; bilateral; ESI: epidural steroid injection; HNP: herniated nucleus pulposus; IL: interlaminar; MB: medial branch; RFA: radiofrequency ablation; SI: sacroiliac; TF: transforaminal; VCF: vertebral compression fracture

Event #	Age	Sex	Augmentation Levels and Procedures	Time Elapsed (weeks)	Intervention Levels and Types	Intervention at Non-Adjacent Levels?	Other Spinal Pathology
1	81	F	L1 & L5 vertebros	2	B/L L3-S1 facet blocks; B/L L4-5 IL ESI	Yes	L2-S1 spinal stenosis, multilevel lumbar facet hypertrophy
2	80	F	L4 & L5 kyphos	9	B/L L3-S1 facet blocks; Left L5-S1 TF ESI	No	L4-5 spondylolisthesis, moderate lumbar spinal stenosis, multilevel spondylosis, L2-3 & L3-4 vacuum discs, scoliosis, L1 VCF
3	84	M	L1 vertebro	10	B/L L1/2 & L5/S1 facet blocks; Left L1/2 TF ESI	Yes	L5-S1 fibrotic disc
4	77	F	T10 & T11 vertebros	12	B/L T9-L1 facet blocks	Yes	Not available
5	69	F	T11 kypho	22	B/L T10-12 facet blocks	No	Scoliosis
6	76	F	T12 vertebro	29	B/L L4-S1 facet/MB RFA	Yes	L4-5 spinal stenosis/ spondylolisthesis/ facet vacuum phenomenon, L1-2 HNP, spondylosis
7*	82	F	T12 kyphos	39	B/L L4-S1 facet blocks; Right L5/S1 TF ESI	Yes	L4-5 spondylolisthesis, L4-5 & L5-S1 vacuum effect, spondylosis
8**	75	F	L4 kypho; L1, L2, L3, & L5 vertebros	48	B/L L3-S1 facet & SI joint blocks	No	Mild lumbar degenerative scoliosis
9*	82	F	L4 & L5 vertebros	53	B/L L4-5 & left L5/S1 facet blocks; Left L4/5 TF ESI	No	L4-5 spondylolisthesis, L4-5 & L5-S1 vacuum effect, spondylosis
10	73	M	L3 & L5 vertebros	61	B/L L3-S3 MB RFA	Yes	Lumbar spondylosis
11	71	F	L4 vertebro	113	B/L L3-5 facet/MB RFA	No	L3-4 anterolisthesis & spinal stenosis, scoliosis, spondylosis
12**	74	F	T9 kypho	187	B/L T9-11 facet/MB RFA & B/L L4-S1 MB RFA	Yes	Mild lumbar degenerative scoliosis
13	88	F	T5 vertebro & T7 kypho	266	B/L L1-4 facet blocks	Yes	T8, T12, L2, & L4 VCFs
14	81	F	T11 vertebro & T12 kypho	304	B/L T10-L2 facet blocks	Yes	Multilevel lumbar spinal stenosis & spondylosis

21% (or 12/56) of the total group of patients reviewed after vertebral augmentation had sufficient pain requiring interventional pain management and fit the study inclusion criteria. The average age of the 12 patients in our inclusion group was 78 years old. 10 patients were female and two were male. 12 patients had one interventional event, while two had two separate events. We performed 25 vertebral augmentation procedures (i.e., 17 vertebroplasties and eight kyphoplasties) on these 12 patients. 15 lumbar, eight lower thoracic, and two mid-thoracic VCFs were augmented. The time from vertebral augmentation procedure to subsequent pain procedure varied from two weeks to over 306 weeks (or 5.8 years). 9/14 cases (66%) of blocks included those performed at non-adjacent levels while 5/14 cases (33%) of blocks were performed only at the same and/or adjacent levels as the VCF. For the events in which thoracic VCFs were augmented only one had a subsequent facet injection for pain adjacent to the thoracic fracture, while 6/7 (or 86%) had developed non-adjacent level pain in areas in the lumbar spine.

The average time elapsed between the patients undergoing vertebral augmentation and subsequent interventional blocks was 83 weeks (1.6 years). Only one case (event #1) had blocks performed within the first six weeks status post-vertebral augmentation. If we consider the entire group of 56 cases, this suggests that significant pain after vertebral augmentation requiring an interventional pain procedure is very uncommon (i.e., 1/56, or less than 2%). In this case, the facet blocks performed included those at non-adjacent levels. A total of 4/12 cases (33%) had a block within 12 weeks of the original vertebral augmentation procedure. Of note is that 3/5 ESIs were performed on the patients who required interventional procedures within the first 10 weeks after the vertebral augmentation. The average pre-procedure visual analog scale (VAS) was eight, while the average post-procedure VAS was 4.5 on their first follow-up visit (two weeks post-procedure) and 2.5 on their second follow-up visit (six weeks post-procedure).

When reviewing other lumbar pathology seen on MRI or CT of these patients at the time of VCF diagnosis, 3/12 had L4-5 degenerative spondylolisthesis, 3/12 had lumbar stenosis (not including patients with L4-5 spondylolisthesis) and 2/12 had degenerative lumbar scoliosis. Therefore, at least 8/12 (67%) had significant spinal stenosis or lumbar degenerative disease. Additionally, 1/12 had L3-4 anterolisthesis and 1/12 had multiple previous lumbar fractures. Thus, in our group, at least 10/12 had pre-existing significant lumbar pathology on MRI/CT scans at the time of fracture treatment. This may have been a major factor in the later development of pain.

## Discussion

In Georgy's study, over a 12 month period, 144 patients underwent vertebral augmentation and 24% of these patients required interventional pain procedures afterwards [1]. In his review, 71% underwent ESI, 18% underwent intercostal nerve blocks, 15% underwent trigger point injections, and 15% underwent sacroiliac joint injections. Most pertinent is that only one patient underwent facet joint blocks, as Georgy did not believe that the facet joints were responsible for the residual pain of VCFs. No conclusions could possibly be drawn about the effect of a facet joint block if only one of these procedures was performed out of 34 patients. Georgy focused predominantly on performing ESIs at non-specified levels, which does not allow for a specific pain generator to be elucidated. In our group, we focused predominantly on facet interventions.

We found a very similar percent, 21%, requiring interventional pain procedures after vertebral augmentation. Less than 2% of the entire group required interventional pain procedures within the first six weeks; however, 86% of interventional blocks after thoracic VCFs were in the lumbar spine. In reviewing imaging at the time of the fracture, 10/12 (83%) had significant lumbar degenerative disease characterized by lumbar stenosis, scoliosis, or spondylolisthesis. Lumbar ESIs are usually indicated in the presence of a herniated disc and/or nerve root compression/inflammation, which may result in radicular pain down the leg. We only found ESIs to be appropriate in 5/14 events, representing patients with spinal stenosis, spondylolisthesis, and/or herniated nucleus pulposus (HNPs). On the contrary, the facet joints are typical generators of axial back pain, which may cause a referred pain. Actually, facet blocks were the mainstay of interventional pain treatment prior to the existence of vertebral augmentation techniques. We performed facet interventions in all 14 events, as the pain represented a more specific axial location and possible pain referral pattern, which was applicable in all 14 events. Our interventions were more focused on targeting specific pain generators based on the location of pain and follow-up CT and MRI scans for more specific results of pain relief.

Pain after a vertebral augmentation procedure could either be residual from the procedure, however new, or increasing pain could develop and needs to be evaluated regarding the known complications of osteoporotic fractures and vertebral augmentation [3]. Gaughen, et al. (2002) believed that pain after vertebroplasty was secondary to an abnormality of the previously augmented VCF [4]. Many mechanisms exist for pain after vertebral augmentation procedures. Same/adjacent level pain may result from vertebral body deformity at the fracture site, cement leakage, fracture progression, a new VCF, facet arthropathy, nerve root entrapment, rib fractures, infection, and non-healing bone-cement interface [5]. In the presence of high-grade pain during the immediate/subacute post-procedure period, specific causes related to the fracture and/or procedure must be ruled out. For the one event in which post-procedure pain developed within two weeks, the pain generators were found to be unrelated to the procedure. The facet joints, both at same/adjacent levels and non-adjacent levels, as well as an L4 spinal nerve, were responsible for the other types of pain. Non-adjacent level pain may result from pre-existing lumbar spine pathology, including lumbar spinal stenosis, spondylosis, spondylolisthesis, and facet arthropathy [5]. Multiple axial structures may contribute to patients’ pain including intervertebral discs, muscles, ligaments, nerves, and fascia. If there is still pain after vertebral augmentation, these pathologies need to be considered, diagnosed and properly treated.

In our group, only one patient out of the 12 required a pain intervention within the first six weeks after vertebral augmentation. This could actually be extrapolated to include all 56 of our patients who sustained VCFs and had vertebral augmentation procedures, as only one out of 56 patients (or less than 2%) had a pain procedure within six weeks after vertebral augmentation. This one patient had multiple interventions, one of which included a non-adjacent level. Out of the remaining cases, all of which took longer than six weeks to undergo subsequent interventions, the majority of them, specifically 9/13 (70%), had interventions performed at non-adjacent levels. The majority of these events had concurrent lumbar spine pathology documented on imaging studies, as noted in Table 1. This suggests that other mechanisms were responsible for the non-adjacent level pain and required time to develop. As noted in our results, 9/12 (75%) had significant spinal stenosis, degenerative spondylolisthesis, or degenerative scoliosis on MRI/CT at the time of fracture treatment. In thoracic VCF augmentation events, 6/7 (86%) developed non-adjacent level pain in the lumbar spine. 

**Figure 1:**
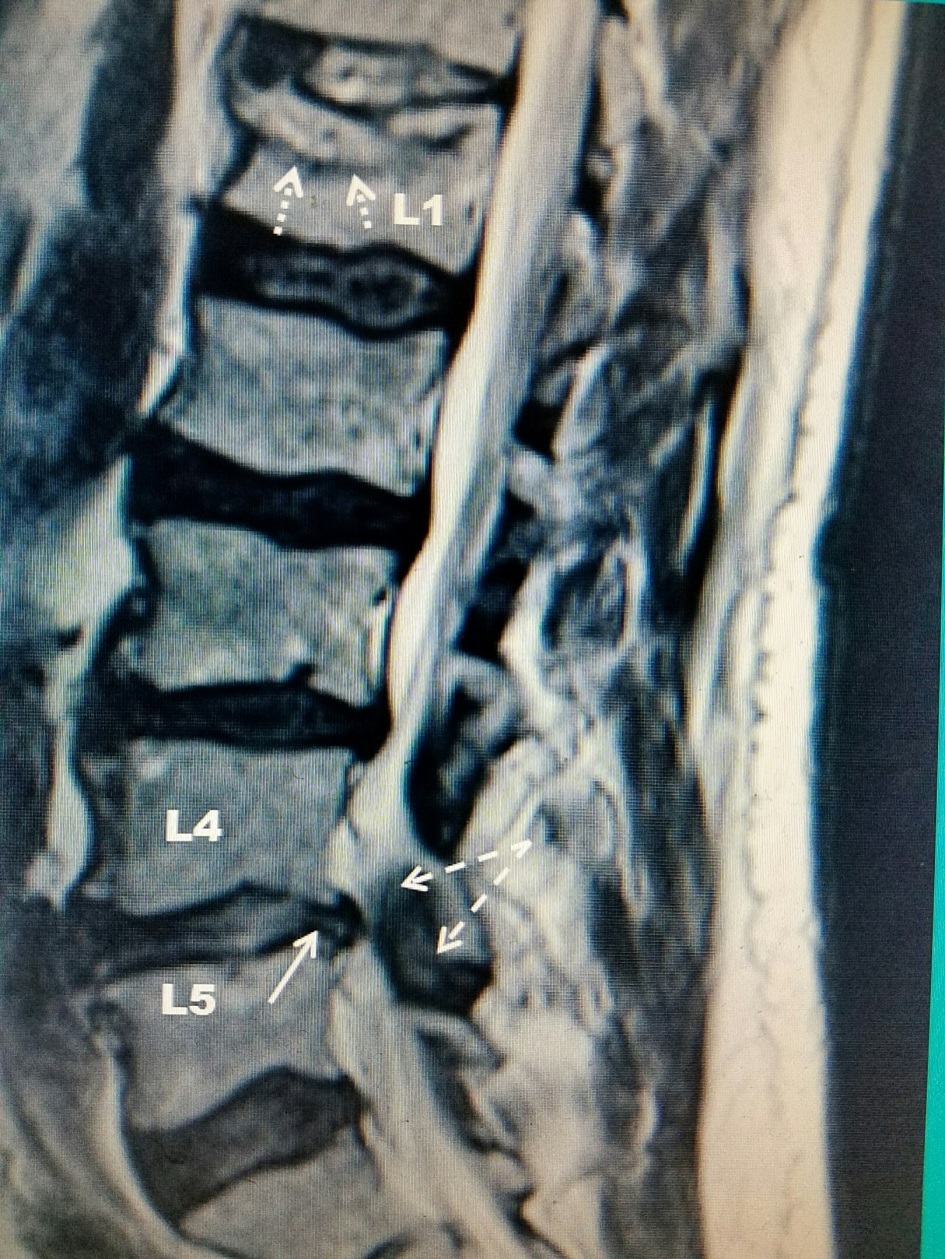
T2 weighted sagittal MRI demonstrating a L1 vertebral compression fracture in addition to L4/L5 central spinal stenosis

Please refer to Figure 1.

Prior to proceding with interventional treatment, conservative treatment is paramount in preventing future injury and pain with VCFs. Physical therapy mainly consists of extension-based exercises to strengthen the surrounding musculature. Flexion of the thoracolumbar spine is typically contraindicated. Huntoon, et al. (2008) demonstrated that patients who underwent vertebral augmentation and subsequently participated in an extension-based strengthening exercise program had a significantly longer median time to re-fracture than those who only underwent vertebral augmentation [6]. Additionally, patients with a VCF who did not undergo vertebral augmentation but participated in the aforementioned exercise program had lower re-fracture rates versus the vertebral augmentation only group. This demonstrates the effectiveness of extension-based exercises in maintaining spinal integrity and the importance of proper biomechanics.

## Conclusions

21% of patients after vertebral augmentation had post-procedure pain that required an interventional pain procedure. This is similar to other previous reports; however, the majority of patients in our study had delayed pain greater than three months after the initial vertebral augmentation that was related to other spinal pathology. If the patient develops pain at a later time, it is often distinguishable from the fracture site pain and, as we show, is usually related to the pre-existing degenerative spondylosis and stenosis rather than the fracture site. If routine conservative treatment, including medications and therapy, is ineffective, this study shows that interventions targeting the facet joints based on the pain location are effective. ESIs with non-specified levels do not allow for the same specificity of interventional pain relief as do facet joint interventions. This study shows that lumbar degenerative changes are closely related to the pain in elderly patients with residual or new pain after previous vertebral augmentation. The facet joints should be the focus of treatment in those patients with known or suspected spinal degeneration.
